# Perception of temporal asymmetries in dynamic facial expressions

**DOI:** 10.3389/fpsyg.2015.01107

**Published:** 2015-08-04

**Authors:** Maren Reinl, Andreas Bartels

**Affiliations:** Vision and Cognition Lab, Centre for Integrative Neuroscience, University of Tübingen, Tübingen, Germany

**Keywords:** faces, perception, fear, emotion, movie

## Abstract

In the current study we examined whether timeline-reversals and emotional direction of dynamic facial expressions affect subjective experience of human observers. We recorded natural movies of faces that increased or decreased their expressions of fear, and played them either in the natural frame order or reversed from last to first frame (reversed timeline). This led to four conditions of increasing or decreasing fear, either following the natural or reversed temporal trajectory of facial dynamics. This 2-by-2 factorial design controlled for visual low-level properties, static visual content, and motion energy across the different factors. It allowed us to examine perceptual consequences that would occur if the timeline trajectory of facial muscle movements during the increase of an emotion are not the exact mirror of the timeline during the decrease. It additionally allowed us to study perceptual differences between increasing and decreasing emotional expressions. Perception of these time-dependent asymmetries have not yet been quantified. We found that three emotional measures, emotional intensity, artificialness of facial movement, and convincingness or plausibility of emotion portrayal, were affected by timeline-reversals as well as by the emotional direction of the facial expressions. Our results imply that natural dynamic facial expressions contain temporal asymmetries, and show that deviations from the natural timeline lead to a reduction of perceived emotional intensity and convincingness, and to an increase of perceived artificialness of the dynamic facial expression. In addition, they show that decreasing facial expressions are judged as less plausible than increasing facial expressions. Our findings are of relevance for both, behavioral as well as neuroimaging studies, as processing and perception are influenced by temporal asymmetries.

## Introduction

Facial expressions are dynamic by nature. It is therefore not surprising that facial motion is a fundamental source of information for social interactions. The importance of motion for face perception has been recognized many years ago (Bassili, 1978; Tomkins, 1982), and several different lines of research have demonstrated that facial motion has indeed facilitative effects on a variety of perceptual and psychological processes. Humphreys et al. (1993) described a patient with visual object agnosia who failed to recognize identity and emotions of static faces, but performed at normal levels when viewing dynamic faces. More recently, facial dynamics have been shown to increase performance on emotion ratings also in healthy participants (Harwood et al., 1999; Wehrle et al., 2000; Ambadar et al., 2005; Biele and Grabowska, 2006; Weyers et al., 2006; Cunningham and Wallraven, 2009) as well as the encoding and recognition of facial identity (Hill and Johnston, 2001; O’Toole et al., 2002; Thornton and Kourtzi, 2002; Knappmeyer et al., 2003; Pilz et al., 2005; Lander et al., 2006). Lederman et al. (2007) showed that dynamic stimuli can improve haptic recognition of emotional faces, and facial dynamics have also been shown to affect physiological automatic responses in that they enhance facial mimicry and affected physiological measures of arousal rates such as heart rate or skin conductance (Simons et al., 1999; Weyers et al., 2006; Sato and Yoshikawa, 2007a).

However, only little is known about possible reasons that drive the perceptual advantage of dynamic face stimuli. Dynamic displays can be described as a series of static images that provide an increased amount of information to the observer, which could be the underlying cause for the dynamic advantage. In Ambadar et al. (2005) tested this hypothesis by presenting either static, multi-static (frames of dynamic stimuli separated by masks to disrupt the percept of coherent motion) or dynamic stimuli of emotional expressions. If an increase of static information would account for the dynamic superiority, one would expect both the multi-static and the dynamic condition to lead to better recognition results. However, this was not the case. Only the dynamic condition improved recognition rates. This suggests that dynamic sequences carry a distinct source of information that is not present in additional static cues. In line with this, Lander and Bruce (2004) observed impairments in identity recognition for scrambled, reversed and decelerated dynamic face movies. Pollick et al. (2003) found that spatial exaggeration of motion trajectories had a substantial effect on recognition rates and intensity ratings of different emotions while temporal variations only lead to small effects on emotion perception. Despite this, humans were shown to be highly sensitive in detecting small changes in the time course of facial movement trajectories (Dobs et al., 2014) and able to reproduce the temporal order of facial expressions from a scrambled set of photographs (Edwards, 1998).

Imaging studies have shown that brain regions responsive to static faces increased their activity in response to facial motion, even when attention was distracted, suggesting that additional neural processes are recruited for processing dynamic compared to static faces (Kilts et al., 2003; Labar et al., 2003; Sato et al., 2004; Fox et al., 2009; Schultz and Pilz, 2009; Trautmann et al., 2009). fMRI also showed a sensitivity of face processing brain regions to the fluidity of facial motion (Schultz et al., 2013), and MEG revealed changes in neural activation for scrambled versus correct-order facial expressions using (Furl et al., 2010). Finally, patient studies showed a dissociation between impairments of static and dynamic facial expressions: PS, a patient with acquired prosopagnosia was impaired in categorizing static facial expressions, but performed normal in categorizing dynamic facial expressions (Richoz et al., 2015).

In sum, it appears that the dynamics, the timing, and the correct temporal sequence of dynamic facial expression changes are crucial for the dynamic face advantage. Thus, directionality is a key aspect in dynamic face processing and perception. This has also been corroborated by computational modeling and theory of visual biological motion processing (Giese and Poggio, 2003). For faces, the importance of directionality is easily illustrated in the example where the direction of change from a neutral to an emotional facial expression (i.e., increasing fear) carries a different ecological meaning than the reversed direction (i.e., relaxing from fear). Increasing fear could, e.g., signal approaching danger to an external observer, whereas relaxing from fear the opposite, even though the average static face information is identical in both conditions. Correspondingly, prior experiments found that the direction of emotional change had perceptual effects, referred to as representational momentum (Freyd and Finke, 1984; Finke and Freyd, 1985). These studies showed differences in subjective rating of facial emotion, e.g., when the intensity of a neutral facial emotion was rated when it was the end-frame of a movie clip starting with a happy or a sad facial expression (Yoshikawa and Sato, 2008; Jellema et al., 2011; Marian and Shimamura, 2013).

Another temporal instance of directionality has received less attention in the past: the sequence of facial movements during relaxation of an emotional expression may not be the exact reverse of the increase of that expression. In prior studies, activation time courses of facial action units showed temporal asymmetries during basic emotional expressions such as happiness or fear (Dobs et al., 2014; Jack et al., 2014). Also, the information content graspable from the face evolves over time: while the earliest components of facial expressions allow for a crude differentiation of approach versus avoidance, the later components signal socially more complex categories (Jack et al., 2014). We hypothesize that unless the temporal evolvement of increasing and relaxing facial expressions is exactly the same, reversing the direction will be perceived differently by a human observer. Such differences will reflect the presence of temporal asymmetry between the two timecourses. In a previous fMRI study we found that face selective regions responded differentially to natural and reversed timelines of dynamic facial expressions, even if controlled for expression direction (i.e., increase and decrease). Since the corresponding static start- and end-frames of these movies did not elicit distinct responses, these brain regions must have been differentially activated due to asymmetries in facial dynamics (Reinl and Bartels, 2014). In addition, we also found neural effects of the expression direction (increasing versus decreasing), partly independent from the timeline manipulation. In the present study we aimed to examine corresponding behavioral effects, i.e., subjective perceptual consequences of natural versus reversed trajectories. To our knowledge, it is not known whether behavioral judgments of human observers are sensitive to these asymmetries and if, how it influences the evaluation of the facial emotions.

Fear is one of the prototypical expressions of high ecological importance that needs to be transmitted and recognized rapidly in order to act efficiently as a warning sign to peers. We therefore chose this a first expression to examine temporal asymmetry. Even though temporal asymmetry should also be studied in a range of other expressions, we would expect similar effects given that they play a role in one of the prototypical expressions.

We first quantified physical motion in our natural face stimuli in order to test whether temporal asymmetries do exist in our stimulus set. Stimuli were genuine movie recordings of increasing and decreasing fearful expressions. These were presented in the natural forward frame order as well as reversed. This led to a 2-by-2 factorial design (increasing versus decreasing facial expression, and natural versus reversed frame order). This allowed us to study behavioral effects of timeline reversal as well as emotional directionality. We hypothesized that the visual system is used to certain natural temporal asymmetries. Deviations, such as induced by timeline reversal, would be reflected in a decrease of perceived emotional intensity, as reversed timelines lead to an atypical unfolding of the emotion. Second, we hypothesized that reversed timelines lead to an increase of perceived artificialness, and to a decrease of convincingness or plausibility of the emotion portrayal.

## Materials and Methods

### Participants

Data were obtained in two separate sessions. The first session was conducted with 28 caucasian participants (15 male, mean age 27 ± 4 years, 1 left-handed). A second session was conducted a few months later to extend the results from the first session. Unfortunately, it was not possible to re-test all subjects of the first session, so the follow-up session included only 19 of the previous 28 subjects. Subjects were healthy with normal or corrected-to-normal vision. The study was conducted according to the declaration of Helsinki and was approved by the local ethics committee of the University of Tübingen. Participants provided written consent prior to participation.

### Stimuli and Procedure

Stimuli consisted of colored short movie clips of eleven caucasian actors portraying fearful facial expressions. Seven movies were recorded prior to the experiment, four movies were selected from the Video-Face-Database of the MPI Tübingen (Kaulard et al., 2012). Actors were asked to show fearful expressions, starting from a neutral face, going to peak expression and relaxing back to a neutral expression. They were asked to keep their head still to minimize rigid head movements. To improve validity of the expression, actors were told to imagine a fearful situation while posing the expression. From every actor several repetitions were recorded. Movies were then selected by visual inspection. Criteria were a recognizable fearful expression with a clear increase and decrease as well no or only little head movement and no excessive eye blinking. Recordings were cut to show either an increase or a decrease of emotional intensity ranging from low to high fear expression or vice versa using VirtualDub (virtualdub.org). Subsequent to this, head-motion was removed by calculating the point-of-gravity (based on luminance values) for each frame and re-centering each frame to its mid-point. The movies were cut at the apex of the expression. The resulting mean durations of both emotional directions (mean and sd: 581 ± 145 ms and 643 ± 245 ms respectively) did not differ statistically [*t*(10) = –0.90, *p* = 0.39]. The means of luminance and of spatial variance for all movies were 96.04 cd/m2 luminance and 109.03 cd/m2 root-mean-square (RMS) contrast, respectively.

**FIGURE 1:**
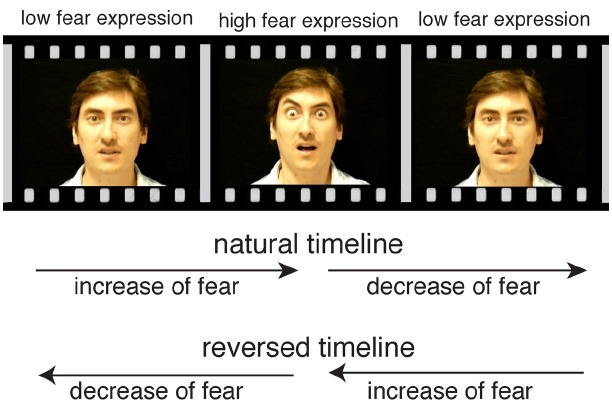
**Illustration of stimulus material.** The four conditions of the experiment were created by playing increasing and decreasing fearful face movies both in a natural as well as in a reversed frame order.

Movies were presented in original and in reversed frame order, giving rise to four conditions: increasing and decreasing fear in original frame order (natural timeline), and decreasing and increasing fear in reversed frame order (reversed timeline), with 11 exemplars for each condition (see Figure 1). Subjects were placed in front of a computer monitor and every movie of each condition was presented to them once in a random sequence that was counterbalanced across subjects. Each presentation was followed by a visual presentation of a scale for rating purposes as described below. Subjects had no time limit for their responses. After their response there was a fixation cross of one second duration before the next stimulus appeared on screen.

### Quantification of Physical Motion in Facial Trajectories

In order to test—in a very basic fashion—whether the dynamic facial expressions in our dataset did indeed contain temporal asymmetries in their motion content, we subjected our stimuli to a computational optic flow algorithm that has been shown to be physiologically plausible (Bülthoff et al., 1989) and that we have successfully used in the past to relate motion in natural movies to brain activation in visual motion regions (Bartels et al., 2008). This algorithm tracks local motion vectors across each frame-pair. Local motion was estimated in a 20-by-20 grid (resulting in 400 vectors) evenly spaced across each frame. The sum of the lengths of all local motion vectors for each frame-pair yielded a time-series of motion intensity for each of the movies. These motion timelines were interpolated to an equal length of 40 time-points (corresponding to 60 Hz for 600 ms long movies), and range-normalized such that all values fell between 0 and 1 for each movie. Two tests were applied to quantify temporal asymmetry. First, we tested for temporal asymmetry within the same movie, i.e., we subtracted motion intensity timelines of forward from reversed movie, and averaged the absolute differences across the different movies, for every time-point. This yielded a time-series of motion difference. This was done separately for increasing fear expression movies (natural vs. reversed timeline) and for decreasing fear movies (natural vs. reversed timelines). Second, we performed the same procedure across increasing fear movies (original timeline) versus reversed timeline for decreasing fear movies, again performed pair-wise within the same actor and summed across actors. T-tests were then applied for every timepoint and Bonferroni-corrected for the number of timepoints (*n* = 40) to identify timepoints where timelines were significantly different between forward and reversed timelines, within or across movies, respectively.

### Rating of Emotional Intensity

In the first session, subjects were asked to rate the amount of fear presented in the movies on a scale from 1 (low fear) to 6 (high fear). Subjects were instructed to indicate the maximum intensity or strength of fear displayed by the facial expression, regardless of the quality or naturalness of the acting.

### Rating of Artificialness and Convincingness

After the intensity rating of the first session revealed significant effects regarding timeline changes, we decided to extend the investigation of perceptual effects and performed a second rating session. In particular, we reckoned apart from intensity, also convincingness and perceived artificialness could be affected by the manipulation. Thus, subjects were subsequently asked to rate two more measures: the artificialness of the actor performance as well as how convincing fear was portrayed by the actors. The reason why we added these two additional measures to our behavioral tests was the following. We assumed that playing movies backward would make the facial dynamics appear unusual, which is tested by the rating of artificialness. Subjects were instructed to rate to which extent they perceived the movement of the face as strange in any way, independent from the quality of the emotion portrayal, its genuineness (i.e., was the actor really feeling the emotion he or she is portraying), or its intensity. After each presentation of a stimuli, they were asked to answer the question “As how natural would you describe the movie?” on scale from 1 (“very natural”) to 8 (“very artificial”).

Second, we hypothesized that the degree to which a dynamic facial expression is judged as plausible emotion portrayal may be affected by our manipulations as well. Note that this rating, which we refer to as “convincingness,” does not necessarily need to be coupled to the artificialness rating, nor to the intensity of the displayed emotion. For example, a strong emotional expression can be perceived as entirely acted and non-genuine (i.e., the actor was not really feeling the emotion). Equally, a well-acted emotional expression can be non-genuine but highly plausible/convincing. The distinction between these ratings has also been discussed previously (Sato and Yoshikawa, 2004; Krumhuber and Kappas, 2005). We asked our subjects to evaluate how convincing or plausible the emotion was portrayed by the actors regardless of whether it seemed to be genuine or not. After each presentation of a stimuli, they were asked to answer the question “How convincing was the emotion fear portrayed?” on a scale from 1 (“very convincing”) to 8 (“not convincing”). Note, that for a better understanding the scores have been reversed in the result figure (Figure 3, results are presented from 1 “not convincing” to 8 “very convincing”).

## Results

**FIGURE 2:**
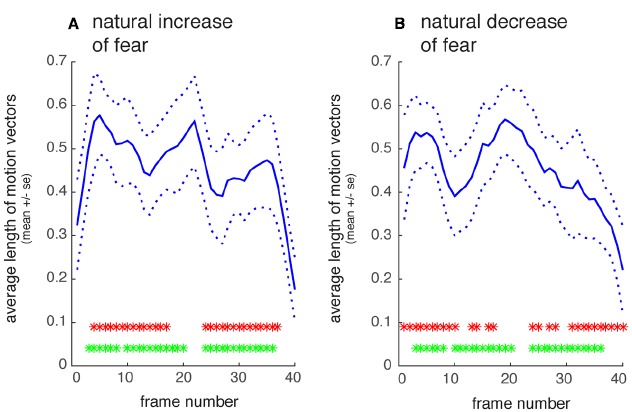
**Quantification of motion intensity over time. (A)** Natural recordings of increasing fear, averaged across 11 actors. **(B)** Natural recordings of decreasing fear. The solid line shows the normalized average length of local motion vectors that were estimated for each frame-pair; dotted line: SE The upper row of asterisks shows time-points where the natural and reversed timelines differ significantly (*p* < 0.05, Bonferroni-corrected for 40 frames) in motion intensity. The lower row of asterisks shows timepoints where increasing fear movies differ in motion from reversed decreasing fear movies (same correction as above). X-axis denotes time in movie-frames (1/60s), y-axis normalized motion intensity.

First, a quantification of motion intensity over time was carried out for each movie in order to obtain a very basic measure of asymmetry of motion content over time. Using the obtained motion-intensity timelines of each movie three tests were carried out. First, we tested for temporal asymmetry within natural recordings of increasing fear, and separately for those of decreasing fear. Figure 2 shows the average timeline of motion intensity for increasing and decreasing fear, respectively. For each of these two emotion directions, we subtracted each individual movie motion timeline from its reversed counterpart to test for temporal asymmetry within a given movie category. For the most part the natural and reversed timelines differed, i.e., each movie category turned out to have asymmetric motion timelines (increasing fear: *T*(11) = 8.05, *p* < 0.001; decreasing fear: *T*(11) = 10.06, *p* < 0.001). Second, we tested whether increasing fear movies were matched in their motion timeline by reversed timelines of decreasing fear movies. Again, for the most part the timelines differed, indicating asymmetry between increasing and decreasing fear expressions (*T*(11) = 15.43, *p* < 0.001). Last, we tested whether the overall amount of asymmetry differed between increasing and decreasing fear movies. This was not the case, i.e., both movie categories were matched in asymmetry (*T*(11) = –0.45, *p* = 0.66).

In the following, we describe subjective ratings obtained using these movie stimuli. Differences for the factor timeline can be attributed to the above observed timeline asymmetries, since static content, overall motion content, and low-level properties were matched across movies and balanced in the factorial design.

Subjective perceptual ratings were obtained from human observers about the convincingness, artificialness and emotional intensity of natural facial expression movies. The movies showed dynamically increasing or decreasing facial expressions of fear, either in natural (forward) frame order or in reversed (backward) frame order.

While each individual rating has ordinal scales of measurement, statistics were carried out using the mean values from each subject for each condition of each rating, i.e., on continuous values. To test whether the resulting mean values follow Normal distributions, we calculated Shapiro-Wilk-Tests for each dataset. The results confirmed that the mean values do not differ from Normal distribution [rating of intensity: W(112) = 0.984, *p* = 0.185; rating of artificialness: W(76) = 0.985, *p* = 0.522; and rating of convincingness: W(76) = 0.975, *p* = 0.131]. Accordingly, parametric testing was used for further analysis: two-by-two ANOVAs with the factors “timeline” (levels: natural, reversed) and “emotion-direction” (levels: increase, decrease) were calculated for each of the rated features.

**FIGURE 3:**
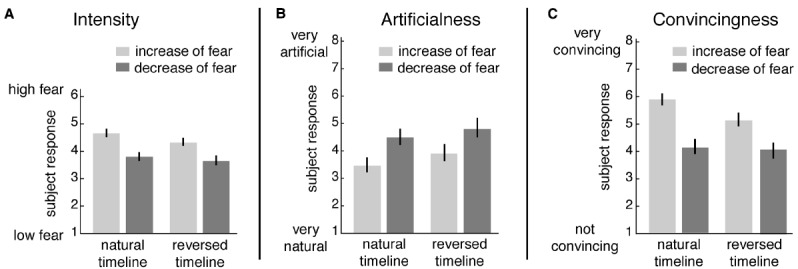
**Behavioral ratings (mean and standard error) of dynamic face stimuli. (A)** Intensity of fear was rated on a scale from 1 to 6 ranging from “1 = low fear” to “6 = high fear,” **(B)** artificialness and **(C)** convincingness were rated on a scale from 1 (“very artificial” or “not convincing”) to 8 (“very natural” or “very convincing”).

The following results were observed. Ratings of emotional intensity (Figure 3A): the ANOVA revealed a main effect of “emotion-direction” [*F*(1,24) = 71.55, *p* < 0.001] and a weaker, yet significant main effect of “timeline” [*F*(1,24) = 17.56, *p* < 0.001]. There was no interaction [*F*(1,24) = 1.7, *p* = 0.194]. Participants rated emotional intensity higher for increasing compared to decreasing fear, and forward played movies were rated more fearful than reversed movies.

Ratings on artificialness (Figure 3B) showed that decreasing fear stimuli were perceived significantly more artificial than increasing fear, and reversed played movies were perceived more artificial than forward played movies [ANOVA: main effect “emotion-direction” *F*(1,14) = 10.25, *p* = 0.005; main effect “timeline” *F*(1,14) = 12.58, *p* = 0.002]. There was no interaction [*F*(1,14) = 0.48, *p* = 0.497].

Similar to the above, ratings of emotional convincingness (Figure 3C) revealed that decreasing fear was perceived as less convincing than increasing fear [ANOVA: main effect “emotion-direction”: *F*(1,14) = 49.64, *p* = 0.001]. Forward played movies were more convincing than reversed movies [ANOVA: main effect “timeline” *F*(1,14) = 16.79, *p* < 0.001]. However, *post hoc*-tests following a significant interaction [ANOVA: “interaction” *F*(1,14) = 39.35, *p* < 0.001] showed that the timeline effect can only be found for increasing but not decreasing fear [*post hoc* paired *t*-tests for the factor “emotion-direction”: “natural increase vs. natural decrease” *T*(18) = –7.89, *p* < 0.001; “artificial increase vs. artificial decrease” *T*(18) = –5.60, *p* < 0.001; *post hoc* paired *t*-tests for the factor “timeline”: “natural increase vs. artificial increase” *T*(18) = –6.02, *p* < 0.001; “natural decrease vs. artificial decrease” *T*(18) = –0.81, *p* = 0.426].

## Discussion

In the current study we examined whether and how time-reversals of dynamic facial expression movies affect subjective experience of human observers. Implicitly, our study also tested whether dynamic facial expressions contain temporal asymmetries, and how they affect perception: if the timeline trajectory of facial action units or of facial muscle movements during the increase of an emotion is not the exact mirror of the timeline during facial relaxation, playing videos in reversed frame order will lead to atypical facial motion trajectories and differences in behavioral ratings. To test this, we recorded natural dynamic facial expressions of increasing and decreasing fear, and played them either forward in the natural frame order (natural timeline) or reversed from last to first frame (reversed timeline). Our design controlled for visual low-level properties, static visual content, and motion energy across the different factors. We found that all three emotional measures, emotional intensity, artificialness and convincingness, were affected by timeline-reversals as well as by the emotional direction of the facial expressions.

### Effects of Timeline

The results on the main effects of timeline show that temporally reversed facial dynamics appeared more artificial, less fearful and, in the case of increasing fear, less convincing to our subjects than the natural timeline counterparts. Previous studies on moving objects (Stone, 1998; Chuang et al., 2005; Schultz et al., 2013; Dobs et al., 2014) already indicated that the visual system is well tuned to temporal statistics. Our results provide direct evidence that human perception is well tuned to the familiar temporal order of muscle movement that occurs during simple emotional fear expressions, and that it detects the fine temporal asymmetry of the sequence of these muscle movements during rise and fall of the expression. One process that may partly account for this could be facial mimicry—the imitation of the facial expression perceived by the viewer. Facial mimicry typically accompanies perception of facial emotions, and it occurs spontaneously and rapidly (Hoffman, 1984; Hatfield et al., 1993). Facial mimicry has been shown to help identifying facial expressions (Niedenthal et al., 2001; Krumhuber and Manstead, 2009; Maringer et al., 2011) and is more pronounced when watching dynamic compared to static expressions (Weyers et al., 2006; Sato and Yoshikawa, 2007b; Sato et al., 2008). As reversed facial movies seem not to follow the usual movement trajectories, facial mimicry might not work properly and may therefore contribute to the perception of the facial expressions as more artificial and less convincing. As natural facial expressions follow non-linear trajectories (Cosker et al., 2010), similar effects have been reported for linear expression morphs, that are also rated as less intense and natural than genuine recordings and that are recognized slower and less accurately (Wallraven et al., 2008; Cosker et al., 2010).

Cunningham and Wallraven (2009) also tested the effects of time-reversal of facial dynamics on the identification of different expressions. They compared recognition rates of movies that were played either in forward or backward frame order. Forward, i.e., natural timelines showed slightly higher recognition rates than reversed timelines, independent of the portrayed expression, emphasizing the importance of temporal direction. However, in contrast to us, they did not control for the change in emotion direction that results from timeline reversal. A study of Hill and Johnston (2001) provided another interesting approach that underlined the impact of facial motion. They animated a standard head with various movement patterns of different people. An identity recognition task indicated that subjects could discriminate the correct identities from only the facial dynamics above chance.

Taken together, those findings prove the importance of facial motion on face perception. Our findings further show that humans are sensitive to effects of temporal direction, also when high-level effects of emotion-direction reversal, as well as low-level and static effects are balanced, and provide an account for enhanced emotion recognition for forward movies provided in previous studies.

### Effects of Emotion-direction

The results on the main effects of emotion-direction show that decreasing fear was rated as less fearful, more artificial and less convincing than increasing fear.

Fear is a very salient stimulus that draws attention as it signals potential danger. Most studies using dynamic stimuli have used frames from neutral to peak expressions (Kessler et al., 2011; Foley et al., 2012), the full course (i.e., increase followed by decrease; Kilts et al., 2003) or morphs (Kamachi et al., 2001; Labar et al., 2003; Biele and Grabowska, 2006; Sato and Aoki, 2006). Morphs do not contain temporal asymmetries, such that decrease equals reversed increase. To our knowledge, no study has explicitly examined genuine isolated relaxation of fear in detail. Sato et al. (2010) used morphs showing increasing and decreasing fear and found lower intensity ratings for decreasing fear, which we confirm here. In addition, we extend their findings to genuine recordings and show effects that reveal timeline asymmetries in genuine recordings.

What possible reasons could have contributed to the less convincing and more artificial appearance of fear decrease? We can offer two potential explanations for these results. First, it is conceivable that it was easier for our actors to act (or imagine getting into a state of) fear, compared to the reverse that is typically less frequently asked of them to do. Second, the difference may lie in the beholders eye: even though in daily life we may be exposed to just parts of a facial expressions (i.e., when turning to someone), we might be more familiar with increasing than decreasing fear as this has a higher salience for us. Observers may be more used to paying attention to increases of emotional expression, making it more likely to rate what they typically pay less attention to as less convincing. The same account may also explain that when playing the movies in reverse, artificially increasing fear is rated less convincing than natural increasing fear while artificially decreasing fear does not seem to differ much from natural decreasing fear.

### Generalization

The question can be raised whether the effects found in our experiment also generalize to other emotional expressions. As mentioned above, Cunningham and Wallraven (2009) did not find any differences between the expressions they tested. However, they point out that some expressions rely more on motion than others. The effects observed in our study clearly depend on the presence of temporal asymmetries in rise and relaxation of the emotional expression. The extent of such asymmetries might vary between different expressions, but this has so far not been systematically quantified in objective or psychometric ways.

The fact that we found relatively robust effects even for one basic emotion expression shows that temporal asymmetries are an important component of facial expressions. This would be of high relevance for both, behavioral as well as neuroimaging studies, as perception and processing are influenced by temporal asymmetries. In particular, asymmetries can by definition only occur in natural movies of facial expressions, but are absent in artificially created linear morphs between two expressions.

There are different theories that try to describe the unfolding of emotional faces. One group comprises discrete-emotion theories (Tomkins, 1982; Ekman, 1992) that define a few basic emotions, each of them coupled to a defined neuromotor program. Once triggered, the expression unfolds completely. However, those theories posit that the action units involved occur in a simultaneous fashion, with similar trajectories and coordinated apexes. In contrast, appraisal theories (Smith and Ellsworth, 1985; Smith, 1989; Ortony and Turner, 1990; Frijda and Tcherkassof, 1997; Smith and Scott, 1997), such as the component-process model of Scherer and Ekman (1984) suggest sequential onsets of action units that can still be modulated during unfolding depending on the situation. Wehrle et al. (2000) aimed to test both types of models and used dynamic stimuli where action units were activated either sequentially or simultaneously but could not find a priority effect for any of them. Our results likewise do not support one theoretical account over the other, but suggest the existence of prototypical unfoldings of emotional expressions that contain temporal asymmetries, at least for fear.

We quantified motion intensity in the facial expression videos, and show significant temporal asymmetries, both within natural recordings of a given emotion-direction, as well across, e.g., natural increasing fear vs. reversed decreasing fear. Our quantification method, however, does not reveal which parts of the faces contains the most asymmetries, and whether asymmetries also exist between rather than within single action units. This interesting question is beyond the scope of the current study and would require analysis of the movement of facial action units based on more detailed facial motion data, e.g., derived using face marker tracking that is not available here.

The importance of facial motion is also evident in neurological or psychiatric disorders. Patients with brain damage, prosopagnostics (Humphreys et al., 1993), the blind (de Gelder et al., 1999), or patients with developmental disorders like autism have been shown to benefit from facial motion in the recognition of emotional expressions and of identity, while failing with static images (Harwood et al., 1999; Back et al., 2007). Mechanisms involved in processing of facial motion trajectories appear to play an important role for these patient groups, making it worthwhile to characterize which facial motion features contribute to the dynamic face advantage in both healthy and patient populations.

### Conflict of Interest Statement

The authors declare that the research was conducted in the absence of any commercial or financial relationships that could be construed as a potential conflict of interest.
